# The *Dermacentor* (Acari, Ixodida, Ixodidae) of Mexico: hosts, geographical distribution and new records

**DOI:** 10.3897/zookeys.569.7221

**Published:** 2016-02-24

**Authors:** Carmen Guzmán-Cornejo, Richard G. Robbins, Alberto A. Guglielmone, Griselda Montiel-Parra, Gerardo Rivas, Tila María Pérez

**Affiliations:** 1 Laboratorio de Acarología, Facultad de Ciencias, Universidad Nacional Autónoma de México, C. P. 04510, D.F. México; 2 Armed Forces Pest Management Board, Office of the Assistant Secretary of Defense for Energy, Installations and Environment, Silver Spring, MD 20910-1202, U.S.A.; 3 Instituto Nacional de Tecnología Agropecuaria, CC 22, C.P. 2300 Rafaela, Santa Fe, Argentina; 4 Colección Nacional de Ácaros, Departamento de Zoología, Instituto de Biología, Universidad Nacional Autónoma de México, C. P. 04510, D.F. México

**Keywords:** *Dermacentor*, ticks, hosts, distribution, Mexico

## Abstract

Distribution and host data from published literature and previously unpublished collection records are provided for all nine species of the Holarctic tick genus *Dermacentor* that are known to occur in Mexico, as well as two species that may occur there. Parasite-host and host-parasite lists are presented, together with a gazetteer of collection localities and their geographical coordinates.

## Introduction

The genus *Dermacentor* Koch, 1844 is a largely Holarctic group of ticks that may be characterized as follows: eyes and festoons present, basis capituli sub-rectangular, palps short and thick, and scutum usually ornate. Most species are three-host parasites of mammals, although two Mexican species, *Dermacentor albipictus* (Packard) and *Dermacentor nitens* Neumann, are one-host ticks. Adults of three-host species usually feed on medium-sized to large mammals, whereas immatures feed on small mammals. This group includes species that are important vectors of microorganisms causing disease in humans and domestic and wild animals (Cooley 1938, Yunker et al. 1986, Durden and Beati 2014).

In the Western Hemisphere, the genus *Dermacentor* currently comprises 14 species, if *Dermacentor kamshadalus* Neumann and *Dermacentor panamensis* Apanaskevich and Bermúdez are included (Guglielmone et al. 2010, Apanaskevich 2013, Apanaskevich and Bermúdez 2013). Collection records for Mexican *Dermacentor* species date to the first half of the 20th century. Hoffmann (1962) and Hoffmann and López-Campos (2000) recognized nine species in this country: *Dermacentor albipictus*, *Dermacentor dissimilis* Cooley, *Dermacentor halli* McIntosh, *Dermacentor hunteri* Bishopp, *Dermacentor imitans* Warburton, *Dermacentor nitens* (formerly classified as both *Anocentor nitens* (Neumann) and *Otocentor nitens* (Neumann)), *Dermacentor occidentalis* Marx, *Dermacentor parumapertus* Neumann, and *Dermacentor variabilis* (Say). Chavarría (1941) stated that *Dermacentor andersoni* occurs in Mexico, but Hoffmann (1962) believed that *Dermacentor andersoni* is not an established Mexican species. Recently, nymphs of *Dermacentor andersoni* were recorded by Gordillo-Pérez et al. (2009) from vegetation in Tamaulipas, but this determination also requires confirmation. A second problematic Mexican species is *Dermacentor latus* Cooley, which was recorded by Cruz-Aldán et al. (2006) and is among the most poorly studied members of this genus (Apanaskevich and Bermúdez 2013). The presence or absence of both *Dermacentor andersoni* and *Dermacentor latus* in Mexico will have to be determined before our inventory of Mexican *Dermacentor* can be considered complete.

## Material and methods

Bibliographic searches were conducted, using an array of public and proprietary databases (Biological Abstracts, BioOne, Biosis, CAB Abstracts, ISI Web of Knowledge), to locate published references to the species of *Dermacentor* that have been reported from Mexico. We then searched the Colección Nacional de Ácaros database (CNAC) (Biota version 1.6.1) to locate any unpublished collection records of Mexican *Dermacentor*. This work is divided into four sections. The first section is a parasite-host list organized alphabetically by tick species and Mexican state. Published tick collection records are presented in the following order: state (capitalized and in **boldface**), collection locality, host species, and reference(s). Where information is unavailable, we denote this as “ND” (Not Determined). For new records, we cite the number and sex or stage(s) (♀ = female, ♂ = male, N = nymph(s), L = larva(e)), locality, date, host name, and CNAC accession number. The second section is a host-parasite list, where hosts and their respective parasites are presented in alphabetical order. Mammalian names have been updated to accord with those of Wilson and Reeder (2005) and Ceballos (2014). The third section is a gazetteer of collection localities and their geographical coordinates. Where coordinates are not available for a specific locality, we reference the coordinates for the nearest municipality. The last section is a map, constructed using the program ArcGIS 9.3 (ESRI 2008), showing the distribution of *Dermacentor* species in Mexico (Fig. 3).

## Results

This work summarizes collection data for 11 *Dermacentor* species known or thought to occur in 31 of Mexico’s 32 federal entities. Mammals belonging to five orders are known to be parasitized by Mexican *Dermacentor*. Although records are provided here for *Dermacentor andersoni* and *Dermacentor latus*, it remains unclear whether these two species occur in the country.

### Parasite-Host List

#### *Dermacentor albipictus* (Packard, 1869)

Figs 1A, 2A

**Records. ND**: east coast of Mexico, horses, asses, mules (Bishopp and Trembley 1945) (referenced as *Dermacentor nigrolineatus*); ND, ND (Vargas 1955); ND, cattle (Becklund 1968) (referenced as *Dermacentor nigrolineatus*). **AGUASCALIENTES**: Asientos, cattle (Hoffmann 1962); ND, horses, deer, cattle (Hoffmann and López-Campos 2000). **BAJA CALIFORNIA**: Unidad de Manejo y Conservación de Vida Silvestre (UMA) “El Tepi,” Sierra San Pedro Mártir, *Odocoileus hemionus fuliginatus* (Contreras et al. 2007). **CAMPECHE**: ND, ND (Hoffmann 1962); ND, horses, deer, cattle (Hoffmann and López-Campos 2000).**CHIAPAS**: Loma Bonita, Selva Lacandona, *Odocoileus virginianus* (Romero-Castañón et al. 2008); Flor de Marqués, Selva Lacandona, *Odocoileus virginianus* (Romero-Castañón et al. 2008); Flor de Marqués, Selva Lacandona, *Mazama americana* (Romero-Castañón et al. 2008); ND, horse (Guglielmone et al. 1990).**CHIHUAHUA**: ND, ND (Woodham et al. 1983). **COAHUILA**: Ocampo, cattle, horses (Chavarría 1941); ND, horses, deer, cattle (Hoffmann and López-Campos 2000). **DISTRITO FEDERAL**: Mexico City, horse (Keirans 1985). **DURANGO**: ND, cattle (Hoffmann 1962); ND, horses, deer, cattle (Hoffmann and López-Campos 2000). **ESTADO DE MÉXICO**: Huehuetoca, cattle, horses (Chavarría 1941); ND, horses, deer, cattle (Hoffmann and López-Campos 2000). **GUERRERO**: Arroyo, Taxco, ND (Hoffmann 1962) (CNAC002100). ND, horses, deer, cattle (Hoffmann and López-Campos 2000). **GUANAJUATO**: ND, ND (Woodham et al. 1983). **HIDALGO**: Hacienda del Astillero, Huichapan, cattle (Hoffmann 1962) (CNAC002102); Sayula, cattle (Hoffmann 1962); Calcali (probably Calnali), ND (Hoffmann 1962) (CNAC002101); ND, horses, deer, cattle (Hoffmann and López-Campos 2000). **JALISCO**: ND, ND (Woodham et al. 1983). **MICHOACÁN**: ND, cattle, horses (Chavarría 1941); ND, horses, deer, cattle (Hoffmann and López-Campos 2000). **NAYARIT**: ND, ND (Woodham et al. 1983). **NUEVO LEÓN**: Sierra de San Antonio Peña Nevada, *Liomys irroratus*, *Peromyscus boylii*, *Peromyscus maniculatus* (Tijerina-Medina et al. 2006). **QUERÉTARO**: ND, ND (Woodham et al. 1983). **PUEBLA**: ND, cattle, horses (Chavarría 1941); ND, horses, deer, cattle (Hoffmann and López-Campos 2000); ND, horses, deer, cattle (Hoffmann and López-Campos 2000). **QUINTANA ROO**: ND, ND (Woodham et al. 1983) (referenced as *Dermacentor nigrolineatus*). **SAN LUIS POTOSÍ**: ND, ND (Woodham et al. 1983). **SONORA**: ND, ND (Woodham et al. 1983). **TABASCO**: ND, ND (Hoffmann 1962); ND, horses, deer, cattle (Hoffmann and López-Campos 2000). **TAMAULIPAS**: ND, ND (Woodham et al. 1983). **VERACRUZ**: ND, cattle, horses (Chavarría 1941); Jilotepec, cattle (Hoffmann 1962); ND, horses, deer, cattle (Hoffmann and López-Campos 2000). **YUCATÁN**: ND, cattle, horses (Chavarría 1941); Temax, ND (Hoffmann 1962); ND, horses, deer, cattle (Hoffmann and López-Campos 2000). **ZACATECAS**: ND, ND (Woodham et al. 1983).

**Figure 1.**
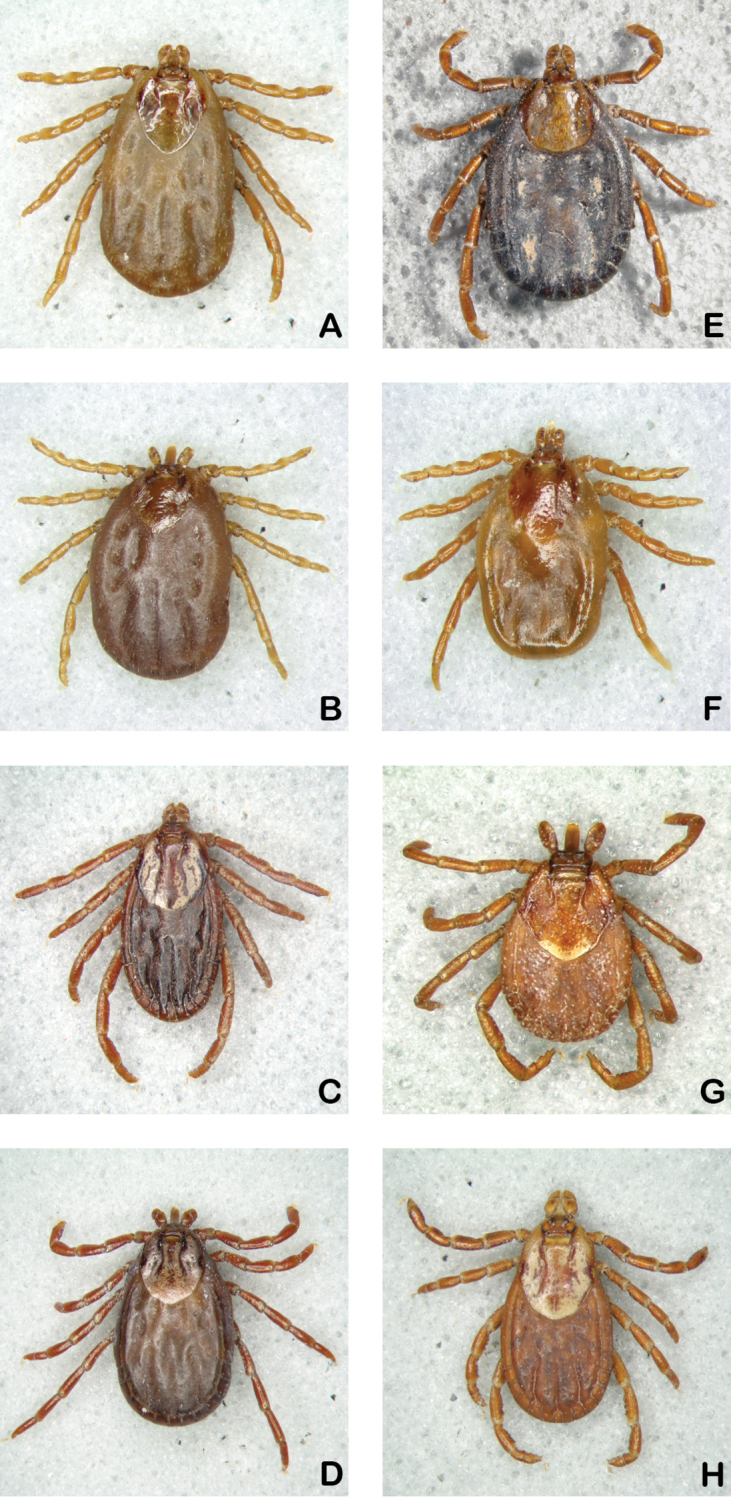
Females. **A** *Dermacentor albipictus* **B** *Dermacentor dissimilis*, **C** *Dermacentor halli* **D** *Dermacentor hunteri* **E** *Dermacentor imitans* **F** *Dermacentor nitens* **G** *Dermacentor parumapertus* **H** *Dermacentor variabilis*.

**Figure 2.**
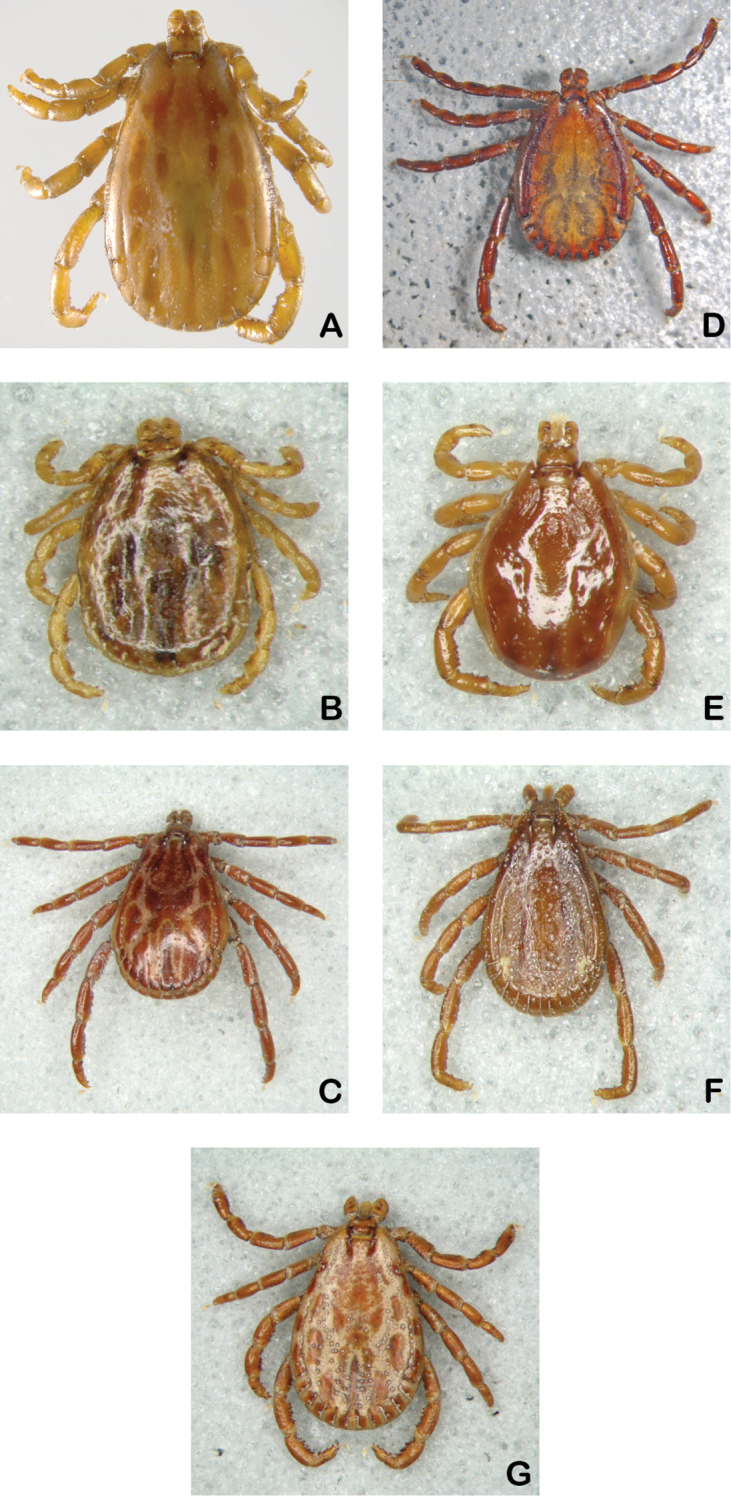
Males. **A** *Dermacentor albipictus* **B** *Dermacentor dissimilis* **C** *Dermacentor hunteri* **D** *Dermacentor imitans* **E** *Dermacentor nitens* **F** *Dermacentor parumapertus* **G** *Dermacentor variabilis*.

**Figure 3.**
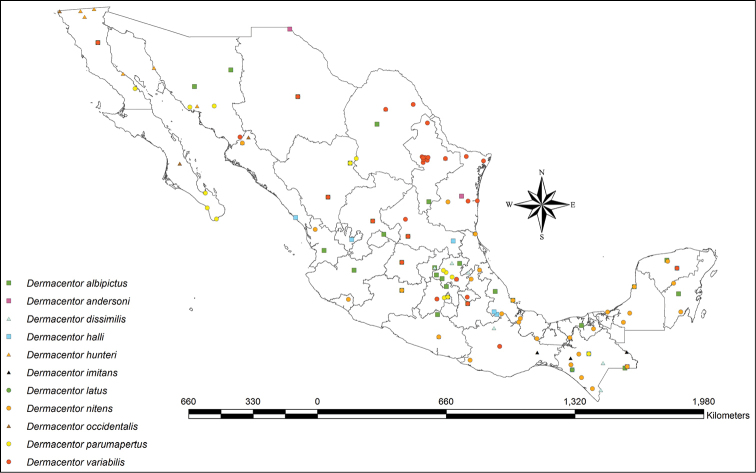
Distribution map of *Dermacentor* species in Mexico. Due to the lack of specific locality data for the states of Guanajuato, Michoacán, Querétaro, Tlaxcala and Zacatecas, all species symbols in those states are solely indicators of occurrence there.

**Notes.** Romero-Castañón et al. (2008) claim that Mexico is a new locality record for *Dermacentor albipictus*, but this species had earlier been recorded from this country by Hoffmann (1962).

**New records. COAHUILA**: 6♀, 3♂, Baca de Huachi, 2-II-1975, cattle (CNAC002105). **VERACRUZ**: 1♀, Jilotepec (CNAC002106).

**Notes.** Baca de Huachi probably refers to Bacadéhuachi; however, this locality is located in Sonora State.

#### *Dermacentor andersoni* Stiles, 1908

**Records. ND**: ND, ND (Vargas, 1955). **CHIAPAS**: Selva Lacandona, *Bos taurus* (Romero-Castañón et al. 2008) (Doubtful record). **CHIHUAHUA**: Ciudad Juárez, sheep (Chavarría 1941). **TAMAULIPAS**: ND, vegetation (Gordillo-Pérez et al. 2009).

#### *Dermacentor dissimilis* Cooley, 1947

Figs 1B, 2B

**Records. ND**: ND, ND (Vargas 1955). **CHIAPAS**: Las Margaritas, about 45 km southeast of Comitán, horses (Cooley 1947); Unión Fronteriza (probably Unión Juárez), horses (Hoffmann 1962) (CNAC002127); ND, horses (Hoffmann and López-Campos 2000). **GUERRERO**: ND, ND (Woodham et al. 1983). **HIDALGO**: ND, ND (Woodham et al. 1983). **MICHOACÁN**: ND, ND (Woodham et al. 1983). **NUEVO LEÓN**: ND, ND (Woodham et al. 1983). **OAXACA**: Teotila, Cuicatlán (probably a locality between Teotitlán and Cuicatlán), horses (Hoffmann 1962) (CNAC002128); ND, horses (Hoffmann and López-Campos 2000). **PUEBLA**: ND, ND (Woodham et al. 1983). **QUERÉTARO**: ND, ND (Woodham et al. 1983). **VERACRUZ**: Zongolica, horses (Kohls and Dalmat 1952); ND, horses (Hoffmann and López-Campos 2000).

**New records. CHIAPAS**: 9♀, 2N, Ciudad Las Casas (probably San Cristóbal de las Casas), VII-1940, horses (CNAC002147). **HIDALGO**: 2♀, Tlahuiltepa, 21-XII-1980 (CNAC002104); 6♀, 1N, San Bartolo Tutotepec, 5-IV-1969 (CNAC002272). **PUEBLA**: 1♀, Puebla, 2-IV-1995 (CNAC002126). **SINALOA**: 1♀, Ocolomé, IX-1944, *Canis familiaris* (CNAC002077). **VERACRUZ**: 4♀, Atescatitla (probably Atexcatitla, Zongolica), 31-I-1946 (CNAC002134).

#### *Dermacentor halli* McIntosh, 1931

Fig. 1C

**Records. ND**: ND, ND, (Vargas 1955); ND, ND (Meleney 1975); **CHIAPAS**: Ciudad Las Casas (probably San Cristóbal de las Casas), dogs (Hoffmann 1962), dogs (Hoffmann and López-Campos 2000); La Sepultura, Reserva de la Biósfera, *Tapirus bairdii* (Cruz-Aldán et al. 2006). **SAN LUIS POTOSÍ**: Taninul, human or vegetation (Fairchild et al. 1966); human, vegetation (Hoffmann and López-Campos 2000). **SINALOA**: Los Pozos, peccary (Fairchild et al. 1966); peccary (Hoffmann and López-Campos 2000).**VERACRUZ**: Atescatitla (probably Atexcatitla, Zongolica), mules (Hoffmann 1962); Zongolica, cattle (Hoffmann 1962); mules, cattle (Hoffmann and López-Campos 2000). **YUCATÁN**: Chichén Itzá (Cooley 1938); mules, cattle (Hoffmann and López-Campos 2000).

**New records. JALISCO**: 1♀, San Buenaventura, El Limón, 17-II- 1997, ND (CNAC005202)

#### *Dermacentor hunteri* Bishopp, 1912

Figs 1D, 2C

**Records. ND**: ND, ND (Vargas 1955). **BAJA CALIFORNIA**: La Rumorosa, human, vegetation, on ground (Williams 1976); ND, *Ovis canadensis* (Hoffmann 1962, Hoffmann and López-Campos 2000); Mexicali, *Ovis canadensis* (Crosbie et al. 1997); Cantil Canyon (probably Cañón Tajo-Cantil), *Ovis canadensis* (Crosbie et al. 1997). **SONORA**: Libertad (probably Puerto Libertad), ND (Cooley 1938); Libertad (probably Puerto Libertad), *Ovis canadensis* (Crosbie et al. 1997); Santa María, *Ovis canadensis* (Crosbie et al. 1997); ND, *Ovis canadensis* (Hoffmann and López-Campos 2000).

**Notes.** The record from Libertad cited by Crosbie et al. (1997) is probably the same record in Cooley (1938).

**New records. BAJA CALIFORNIA**: 1♀, 78♂, Sierra de Camulaje (probably Sierra de Calamajué), 4-III-1974, *Ovis canadensis* (CNAC002136); 3♀, 17♂, ND, “wild sheep” (probably *Ovis canadensis*) (CNAC002136).

#### *Dermacentor imitans* Warburton, 1933

Figs 1E, 2D

**Records. CHIAPAS**: Selvas de El Ocote, Ocozocoautla, *Pecari tajacu*, *Tayassu pecari*, *Mazama americana sartorii* (Hoffmann 1962, Fairchild et al. 1966).

**Notes.** *Mazama satorii* is considered a junior synonym of *Mazama temama* by Wilson and Reeder (2005), but Ramírez-Pulido et al. (2005) classify *Mazama satorii* as a subspecies of *Mazama americana*.

The records of Hoffmann (1962) and Fairchild et al. (1966) are identical – both reference the same RML collection numbers.

**New records. CHIAPAS**: 1♂, Ocosingo, Frontera Corozal, Área Natural Protegida Lacandona, 12-X-2004, vegetation (CNAC005194). **OAXACA**: 1♀, Istmo de Tehuantepec, ND, okapi (sic) (CNAC005018).

#### *Dermacentor latus* Cooley, 1937

**Record. CHIAPAS**: La Sepultura, Reserva de la Biósfera, *Tapirus bairdii* (Cruz-Aldán et al., 2006).

#### *Dermacentor nitens* Neumann, 1897

Figs 1F, 2E

**Records. ND**: ND, ND (Hooker et al. 1912); east coast of Mexico, horses, asses, mules (Bishopp and Trembley 1945); ND, horses (Becklund 1968). **CAMPECHE**: ND, horses, cattle (Chavarría 1941); ND, cattle, horses, donkeys, mules, dogs (Hoffmann 1961); Rancho el Paraíso, cattle (Hoffmann 1962) (CNAC005176). **CHIAPAS**: ND, horses, cattle (Chavarría 1941); ND, cattle, horses, donkeys, mules, dogs (Hoffmann 1961); Huixtla, cattle (Hoffmann 1962); El Vergel (there are two localities with this name, located in two different municipalities: Chiapa de Corzo and Pijijiapan), horses (Hoffmann 1962); Ciudad las Casas (probably San Cristóbal de las Casas), horses (Hoffmann 1962); Zoológico regional “Miguel Álvarez del Toro,” *Tapirus bairdii* (Cruz-Aldán et al. 2006); Flor de Marqués, Selva Lacandona (Romero-Castañón et al. 2008). **CHIHUAHUA**: ND, ND (Woodham et al. 1983). **COLIMA**: ND, horses, cattle (Chavarría 1941); ND, cattle, horses, donkeys, mules, dogs (Hoffmann 1961); Colima, cattle (Hoffmann 1962). **DISTRITO FEDERAL**: near Mexico City, cattle, horse, sheep (Keirans 1985). **DURANGO**: ND, horses, sheep (Chavarría 1941); ND, cattle, horses, donkeys, mules, dogs (Hoffmann 1961). **ESTADO DE MÉXICO**: ND, horses, cattle (Chavarría 1941); ND, cattle, horses, donkeys, mules, dogs (Hoffmann 1961). **GUERRERO**: ND, horses, cattle (Chavarría 1941); ND, cattle, horses, donkeys, mules, dogs (Hoffmann 1961). **HIDALGO**: ND, horses, cattle (Chavarría 1941); ND, cattle, horses, donkeys, mules, dogs (Hoffmann 1961). **MICHOACÁN**: ND, horses, cattle (Chavarría 1941); ND, cattle, horses, donkeys, mules, dogs (Hoffmann 1961). **OAXACA**: ND, horses, cattle (Chavarría 1941); ND, cattle, horses, donkeys, mules, dogs (Hoffmann 1961); Jesús Carranza (on the border of Veracruz and Oaxaca but situated in Veracruz State), ND (Keirans 1985). **PUEBLA**: ND, horses, cattle (Chavarría 1941); ND, cattle, horses, donkeys, mules, dogs (Hoffmann 1961). **QUINTANA ROO**: southeast of Peto, horses (Bequaert 1933). **SAN LUIS POTOSÍ**: ND, horses, cattle (Chavarría 1941); ND, cattle, horses, donkeys, mules, dogs (Hoffmann 1961). **TAMAULIPAS**: Ciudad Victoria, horse, donkey, mule (Hooker et al. 1912, Macías-Valadez 1923); Tampico, horse, donkey, mule (Hooker et al. 1912, Macías-Valadez 1923); Laguna Madre, horse (Drummond and Graham 1964). **VERACRUZ**: ND, horses, cattle (Chavarría 1941); ND, cattle, horses, donkeys, mules, dogs (Hoffmann 1961). **YUCATÁN**: ND, horses, cattle (Chavarría 1941); ND, cattle, horses, donkeys, mules, dogs (Hoffmann 1961).

**New records. CAMPECHE**: 26♀, Candelaria, XI-1944, horse (CNAC005127); 17♀, Escárcega, XII-1944, horse (CNAC005093); 21♀, 1♂, 1N, Escárcega, XII-1944, horse (CNAC005126). **CHIAPAS**: 54♀, 44♂, Villa Flores (probably Villaflores), 6-XII-1983, horse (CNAC002087); 3♂, Rancho Agua Escondida, Villa Flores (probably Villaflores), 5-XII-1983, horse (CNAC002079). **NAYARIT**: 1♀, Acapareta (probably Acaponeta), 24-V-1981, horse (CNAC002295). **OAXACA**: 16♀, 14♂, 8N, Tlacamana (probably Tlacamama), 21-IV-1975, horse (CNAC002085); 7♀, 5♂, Cosolapa, VI-1950 (CNAC002081). **PUEBLA**: 6♀, 5♂, 2L, Huauchinango, VI-1927 (CNAC002083). **QUINTANA ROO**: 4♀, 11♂, Bacalar, VIII-1939, “tepezcuintle” (probably *Cuniculus paca*) (CNAC002088). **SINALOA**: 1♀, Ocolomé, IX-1944, *Canis familiaris* (CNAC002080). **TABASCO**: 26♀, 2 ♂, Macuspana, II- 1971, horses (CNAC002076); 3♂, Amaicote, 26-III-1971, horses (CNAC002266); 4♀, Amaicote, 26-III-1971, horses (CNAC002298). **TAMAULIPAS**: 1♀, 1♂, Rancho la Bolsa, Tampico (CNAC005158). **VERACRUZ**: 4♀, 3♂, Tuxtilla, IX-1939 (CNAC002086); 26♀, 16♂, 8N, Cosamaloapan, IX-1939, *Canis familiaris* (CNAC002082); 6♀, 2♂, Veracruz, VII-1927 (CNAC002078); 3♀, Miahuapa (probably San Pedro Miahuapan), 14-IV-1949, deer (CNAC005083); 3♀, 11♂, Miahuapa (probably San Pedro Miahuapan), 14-XI-1949, horse (CNAC005054).

#### *Dermacentor occidentalis* Marx, 1892

**Records. BAJA CALIFORNIA**: Tijuana, cattle (Secretaria de Agricultura y Fomento de México, 1926, 1930 in Hoffmann 1962); ND, ND (Woodham et al. 1983); Unidad de Manejo y Conservación de Vida Silvestre (UMA) “El Tepi,” Sierra San Pedro Mátir, *Odocoileus hemionus fuliginatus* (Contreras et al. 2007); ND, *Bos taurus* (Hoffmann and López-Campos 2000). **BAJA CALIFORNIA SUR**: ND, ND (Woodham et al. 1983). **COAHUILA**: ND, ND (Woodham et al. 1983). **SINALOA**: Choix (Hoffmann 1925); ND, *Bos taurus* (Hoffmann and López-Campos 2000).

#### *Dermacentor parumapertus* Neumann, 1901

Figs 1G, 2F

**Records. ND**: ND, ND (Vargas 1955); ND, ND (Becklund 1968). **BAJA CALIFORNIA**: Bahía de los Ángeles, *Lepus californicus* (Ryckman and Ryckman 1963); ND, rabbits, hares (Hoffmann 1961). **BAJA CALIFORNIA SUR**: La Paz, *Lepus californicus* (Hoffmann 1962); Todos Santos, rabbits (Hoffmann 1962) (CNAC002144); Puerto Chileno, hare (Hoffmann 1962) (CNAC002146). **CHIAPAS**: Ciudad Las Casas (probably San Cristóbal de las Casas), horses (Hoffmann 1962); ND, rabbits, hares (Hoffmann and López-Campos 2000). **CHIHUAHUA**: ND, cattle (Strickland and Gerrish 1965). **COAHUILA**: Región Lagunera, hares (Silva-Goytia and Elizondo 1952); ND, rabbits, hares (Hoffmann and López-Campos 2000). **DISTRITO FEDERAL**: ND, rabbits, hares (Hoffmann 1961, Hoffmann and López-Campos 2000); Camino al Desierto de los Leones, rabbits (Hoffmann 1962). **DURANGO**: Región Lagunera, hares (Silva-Goytia and Elizondo 1952); ND, rabbits, hares (Hoffmann and López-Campos 2000). **HIDALGO**: Ixmiquilpan, hares (Tovar 1944); ND, rabbits, hares (Hoffmann 1961; Hoffmann and López-Campos 2000); Actopan, rabbits (Hoffmann 1962) (CNAC002145); Taxquillo, rabbits (Hoffmann 1962) (CNAC002148). **SAN LUIS POTOSÍ**: San Luis Potosí, jack rabbits (Roberts 1934). **SONORA**: Cumuripa, hares (Hoffmann 1962) (CNAC002143); Guaymas, hares (Hoffmann 1962); ND, rabbits, hares (Hoffmann and López-Campos 2000).

**New records. DURANGO**: 2♀, 2♂, Ejido 18 de Marzo, Durango, 16-VIII- 1976, hare (CNAC002149). **DISTRITO FEDERAL**: 1♀, 1♂, México D.F., rabbit (CNAC002141). **SONORA**: 1♀, 1♂, Guaymas, 15-VII-1924, hare (CNAC002140).

**Note.** In Hoffmann (1962), the record for Sonora: Guaymas is identical to our new record, except that the year is stated to be 1944, whereas the year on our collection label is 1924, and for that reason we consider our record to be different.

#### *Dermacentor variabilis* (Say, 1821)

Figs 1H, 2G

**Records. ND**: ND, ND (Neumann 1901); ND, ND (Hooker 1909); ND, ND (Hooker et al. 1912); ND, ND (Pinto 1930); ND, ND (Bishop and Trembley 1945); ND, ND (Vargas 1955). **BAJA CALIFORNIA**: ND, ND (Woodham et al. 1983); Unidad de Manejo y Conservación de Vida Silvestre (UMA) “El Tepi,” Sierra San Pedro Mártir, *Odocoileus hemionus fuliginatus* (Contreras et al. 2007). **CHIAPAS**: ND, ND (Tovar 1945); cattle (Hoffmann 1961); Valle Central (Ortega-Gutiérrez 1979); ND, rabbits (Hoffmann and López-Campos 2000). **CHIHUAHUA**: ND, ND (Woodham et al. 1983). **COAHUILA**: ND, ND (Woodham et al. 1983). **DURANGO**: ND, ND (Woodham et al. 1983). **ESTADO DE MÉXICO**: ND, ND (Tovar 1945); cattle (Hoffmann 1961; Hoffmann and López-Campos 2000); ND, ear canal of goats (Hoffmann and López-Campos 2000). **GUANAJUATO**: ND, *Lepus callotis* (Neumann 1901). **HIDALGO**: ND, ND (Tovar 1945); cattle (Hoffmann 1961, Hoffmann 1962, Hoffmann and López-Campos 2000). **NUEVO LEÓN**: ND, ND (Woodham et al. 1983); ND, environment (Oliveira et al. 2010); Guadalupe, dog (Galaviz-Silva et al. 2013); Pesquería, dogs (Galaviz-Silva et al. 2013); Benito Juárez, dogs (Galaviz-Silva et al. 2013); Apodaca, dogs (Galaviz-Silva et al. 2013); Estanzuela, dogs (Galaviz-Silva et al. 2013); Guadalupe, dogs (Galaviz-Silva et al. 2013); Escobedo, dogs (Galaviz-Silva et al. 2013); San Nicolás de los Garza, dogs (Galaviz-Silva et al. 2013). **SAN LUIS POTOSÍ**: ND, ND (Tovar 1945). **OAXACA**: ND, ND (Tovar 1945); cattle (Hoffmann 1961, Hoffmann and López-Campos 2000). **PUEBLA**: ND, ND (Hoffmann 1962). **SAN LUIS POTOSÍ**: cattle (Hoffmann 1961); Venado, cattle (Hoffmann 1962); ND, deer (Hoffmann and López-Campos 2000). **SONORA**: cattle (Hoffmann 1961); El Maquipo, hares (Hoffmann 1962); ND, hares (Hoffmann and López-Campos 2000). **TAMAULIPAS**: Soto La Marina, Rancho La Pesca (Chavarría 1941); Soto La Marina, Hacienda Espíritu Santo (Chavarría 1941); ND, ND (Tovar 1945); cattle (Hoffmann 1961, Hoffmann and López-Campos 2000). **TLAXCALA**: ND, ND (Hoffmann 1962); ND, hares (Hoffmann and López-Campos 2000). **YUCATÁN**: Chichén Itzá, vegetation (Bequaert 1933); cattle (Hoffmann 1961, Hoffmann and López-Campos 2000). **ZACATECAS**: ND, ND (Woodham et al. 1983).

**New records. COAHUILA**: 1♀, 1♂, San Patricio, Villa Unión, 19-V-1975, bovine (CNAC002152). **NUEVO LEÓN**: 2♀, 3♂, Anahuac, 26-VI-1976, wildcat (CNAC002151). **TAMAULIPAS**: 2♀, 1♂, Los tres Garcia, Reynosa, 26-VIII-1976, *Canis familiaris* (CNAC002159); 1♀, 1♂ Matamoros, 19-IV-1999, *Lynx rufus* (CNAC002240).

### Host-parasite List

**Vegetation**

*Dermacentor halli*

*Dermacentor hunteri*

*Dermacentor imitans*

*Dermacentor variabilis*

**Artiodactyla**

**Deer**

*Dermacentor albipictus*

**Goats**

*Dermacentor variabilis*

**Sheep**

*Dermacentor nitens*

**Wild sheep**

*Dermacentor hunteri*

**Peccary**

*Dermacentor halli*

***Bos taurus* Linnaeus (Aurochs, Cattle, Bovine)**

*Dermacentor albipictus*

*Dermacentor halli*

*Dermacentor nitens*

*Dermacentor occidentalis*

*Dermacentor parumapertus*

*Dermacentor variabilis*

***Mazama americana* (Erxleben) (South American Red Brocket)**

*Dermacentor albipictus*

*Dermacentor nitens*

***Mazama americana temama* (Kerr)**

*Dermacentor imitans*

***Odocoileus hemionus fuliginatus* Cowan (Southern Mule Deer)**

*Dermacentor albipictus*

*Dermacentor nitens*

*Dermacentor variabilis*

***Odocoileus virginianus* (Zimmermann) (White-tailed Deer)**

*Dermacentor albipictus*

***Ovis canadensis* Shaw (Bighorn Sheep)**

*Dermacentor hunteri*

***Pecari tajacu* (Linnaeus) (Collared Peccary)**

*Dermacentor imitans*

***Tayassu pecari* (Link) (White-lipped Peccary)**

*Dermacentor imitans*

**Carnivora**

***Canis familiaris* Linnaeus (domestic dog)**

*Dermacentor dissimilis*

*Dermacentor halli*

*Dermacentor nitens*

*Dermacentor variabilis*

***Lynx rufus* (Schreber) (Bobcat)**

*Dermacentor variabilis*

**Lagomorpha**

**Hares**

*Dermacentor parumapertus*

*Dermacentor variabilis*

**Rabbits**

*Dermacentor parumapertus*

*Dermacentor variabilis*

***Lepus californicus* Gray (Black-tailed Jackrabbit)**

*Dermacentor parumapertus*

***Lepus callotis* Wagler (White-sided Jackrabbit)**

*Dermacentor variabilis*

**Perissodactyla**

**Mules**

*Dermacentor albipictus*

*Dermacentor halli*

*Dermacentor nitens*

***Equus asinus* Linnaeus (ass, donkey)**

*Dermacentor albipictus*

*Dermacentor nitens*

***Equus caballus* Linnaeus (horse)**

*Dermacentor albipictus*

*Dermacentor dissimilis*

*Dermacentor nitens*

*Dermacentor parumapertus*

***Tapirus bairdii* (Gill) (Baird’s Tapir)**

*Dermacentor halli*

*Dermacentor nitens*

**Primates**

***Homo sapiens* Linnaeus (human)**

*Dermacentor halli*

*Dermacentor hunteri*

**Rodentia**

***Cuniculus paca* (Linnaeus) (Tepexcuintle, Lowland Paca)**

*Dermacentor nitens*

***Liomys irroratus* (Gray) (Mexican Spiny Pocket Mouse)**

*Dermacentor albipictus*

***Peromyscus boylii* (Baird) (Brush Deermouse)**

*Dermacentor albipictus*

***Peromyscus maniculatus* (Wagner) (North American Deermouse)**

*Dermacentor albipictus*

## List of localities

**Table:** List of localities

	Latitude N	Longitude W
East coast of Mexico	ND	ND
**AGUASCALIENTES**		
Asientos	22°14'18.69"	102°5'21.92"
**BAJA CALIFORNIA**		
Cantil Canyon (probably Canón Tajo-Cantil)	32°15'50"	115°52'54"
Bahía de los Ángeles	28°57'5.07"	113°33'36.11"
La Rumorosa	32°31'37.93"	116°4'15.86"
Sierra de Camulaje (probably Sierra de Calamajué)	29°38'13"	114°6'39"
Tijuana	32°30'53.73"	117°2'18.37"
Unidad de Manejo y Conservación de Vida Silvestre (UMA) “El Tepi” Sierra San Pedro Mártir	31°04'36"	115°16 ’ 31’’
Mexicali	32°37'26"	115°27'5"
**BAJA CALIFORNIA SUR**		
La Paz	24°8'33.28"	110°18'46.86"
Puerto Chileno	22°56'51"	109°48'27"
Todos Santos	23°27'23.07"	110°13'49.04"
**CAMPECHE**		
Candelaria	18°11'30.08"	91°2'28.68"
Campeche	19°49'49.98"	90°32'4.42"
Escárcega	18°36'32.14"	90°44'46.2"
Rancho el Paraíso	18°39'38.23"	91°46'19.84"
**CHIAPAS**		
Ciudad Las Casas (probably San Cristóbal de las Casas)	16°44'12"	92°38'18"
El Vergel (there are two localities with this name, located in two different municipalities: Chiapa de Corzo and Pijijiapan)		
El Vergel, Chiapa de Corzo	16°39'6"	93°00'47"
El Vergel, Pijijiapan	15°38'33"	92°58'21"
Flor de Marqués, Selva Lacandona	16°09'	90°52'
Huixtla	15°8'15.9"	92°27'57"
La Sepultura, Reserva de la Biosfera	16°00" and 16°29"	93°24" and 94°07"
Las Margaritas, abouth 45 km south Comitán	16°19'0"	91°58'57"
Loma Bonita, Selva Lacandona	16°05'	90°58'
Ocosingo Frontera Corozal, Área natural protegida Lacandona	16°49'16"	90°53'25"
Rancho Agua Escondida, Villa Flores (probably Villaflores)	16°14'4.01"	93°27'31.03"
Selva Lacandona	ND	ND
Selvas de El Ocote Ocozocoautla	16°31'56"	93°28'31"
Unión Fronteriza (probably Unión Juárez)	15°4'0"	92°5'0"
Valle Central	ND	ND
Villa Flores (probably Villaflores)	16°14'4.01"	93°27'31.03"
Zoológico regional “Miguel Álvarez del Toro”	16°43'30"	93°5'38.1"
**CHIHUAHUA**		
Ciudad Juárez	31°41'28.48"	106°25'28.2"
**COAHUILA**		
Baca de Huachi (probably Bacadéhuachi in Sonora State)	29°48'35"	109°8'28"
Ocampo	27.316261	102.405747
Región Lagunera	ND	ND
San Patricio, Villa Unión	28°13'25"	100°43'47"
**COLIMA**		
Colima	19°14'42.7"	103°43'28"
**DISTRITO FEDERAL**		
Camino al Desierto de los Leones	19°19'1.6"	99°18'20.74"
Near Mexico City	ND	ND
Mexico City	19°21'11"	99°8'14"
**DURANGO**		
Ejido 18 de Marzo	25°43'54.4"	103°21'28.3"
Región Lagunera	ND	ND
**ESTADO DE MÉXICO**		
Huehuetoca	19°50'5.75"	99°12'11.09"
**GUERRERO**		
Arroyo, Taxco	18°32'33.03"	99°36'47.86"
**GUANAJUATO**		
	ND	ND
**HIDALGO**		
Actopan	20°16'27.58"	98°56'17.44"
Calcali (probably Calnali)	20°53'57.12"	98°35'19.1"
Hacienda del Astillero, Huichapan	20°22'16.78"	99°39'39.45"
Ixmiquilpan	20°29'03"	99°13'08"
San Bartolo Tutotepec	20°29'1.64"	98°11'41.82"
Sayula	20°12'3"	99°24'1"
Taxquillo	20°34'32.98"	99°20'31.34"
Tlahuiltepa	20°55'26.65"	98°57'2.26"
**JALISCO**		
San Buenaventura, El Limón	21°59'48.98"	103°34'12.97"
**MICHOACÁN**		
	ND	ND
**NAYARIT**		
Acapareta (Acaponeta)	22°27'52.2"	105 14 55.89"
**NUEVO LEÓN**		
Anáhuac	27°22'29.56"	100°4'47.74"
Apodaca	25°47'00"	100°11'00"
Benito Juárez	25°39'00"	100°05'00"
Escobedo	25°48'30"	100°19'36"
Estanzuela	25°32'60"	100°16'15"
Guadalupe	25°40'39"	100°15'35"
Nicolás de los Garza	25°46'00"	100°17'00"
Pesquería	25°47'00"	100°3'00"
San Antonio Peña Nevada	23°44'38.99"	101°0'36"
**OAXACA**		
Cosolapa	18°35'2.65"	96°39'11.3"
Teotila, Cuicatlán (probably a road between Teotitlán and Cuicatlán)	17°55'33"	97°0'21"
Istmo de Tehuantepec	ND	ND
Oaxaca	17°5'00"	96°45'00"
Tlacamana (probably Tlacamama)	16°26'48.51"	98°6'42.73"
**PUEBLA**		
Huauchinango	20°10'30.14"	98°3'42.76"
Puebla	19°3'5"	98°13'4"
**QUERÉTARO**		
	ND	ND
**QUINTANA ROO**		
Bacalar	18°40'18.84"	88°23'53.62"
Southeast of Peto	19°59'11"	88°43'14"
**SAN LUIS POTOSÍ**		
San Luis Potosí	22°08'59"	100°58'30"
Taninul	21°56'09"	98°53'19"
Venado	22°56'00"	101°5'34"
**SINALOA**		
Choix	26°42'36"	108°19'34"
Los Pozos	23°00'40"	106°9'12"
Ocolomé	26°26'50.81"	108°36'30.67"
**SONORA**		
Baca de Huachi (probably Bacadéhuachi in Sonora State)	29°48'35"	109°8'28"
Cumuripa	28°9'11.41"	109°54'35.06"
El Maquipo	26°43'35"	108°43'10"
Guaymas	28°6'10.8"	111°1'47.81"
Libertad (probably Puerto Libertad)	29°54'15"	112°40'59"
Santa María	28°8'30"	110°41'35"
**TABASCO**		
Amaicote	17°29'5.2"	93°30'41.32"
Macuspana	17°53'13.27"	92°25'11.42"
**TAMAULIPAS**		
Ciudad Victoria	23°44'00"	99°8'00"
Hacienda Espíritu Santo, Soto La Marina	23°46'8"	98°12'19"
Laguna Madre	ND	ND
Los Tres García, Reynosa	25°49'36.15"	98°17'6.03"
Matamoros	25°37'7.93"	97°29'18.56"
Rancho la Bolsa, Tampico	22°15'57.34"	97°52'24.99"
Rancho La Pesca, Soto La Marina	23°47'16"	97°46'30"
Tampico	22°15'19"	97°52'7"
**TLAXCALA**		
	ND	ND
**VERACRUZ**		
Atescatitla (probably Atexcatitla, Zongolica)	18°33'25"	96°52'46"
Cosamaloapan	18°22'0.8"	95°47'40.77"
Jesús Carranza	17°26'06"	95°1'44"
Jilotepec	19°36'41"	96°56'58"
Miahuapa (probably San Pedro Miahuapan)	20°35'40.12"	97°40'18.58"
Tuxtilla	18°11'43.43"	95°51'54.75"
Veracruz	19°11'57"	96°8'16"
Zongolica	18°40'17.54"	97°0'5.22"
**YUCATÁN**		
Chichen Itzá	20°40'59"	88°34'07"
Temax	21°2'55"	89°2'20"
**ZACATECAS**		
	ND	ND

## Discussion

The first species records of the genus *Dermacentor* in Mexico were made by Hooker (1909) and Hooker et al. (1912), who referenced Mexican specimens of *Dermacentor variabilis* and *Dermacentor nitens*, two species of veterinary importance. The next species recorded from this country was *Dermacentor occidentalis*, cited by Hoffmann (1925) from Choix, Sinaloa. During the 1930s and 1940s, *Dermacentor albipictus*, *Dermacentor dissimilis*, *Dermacentor halli*, *Dermacentor hunteri*, and *Dermacentor parumapertus* were recorded from Mexico for the first time. Most recently, records have been published for *Dermacentor andersoni* (Vargas 1955), *Dermacentor imitans* (Hoffmann, 1962), and *Dermacentor latus* (Cruz-Aldán et al. 2006).

According to Apanaskevich and Bermúdez (2013), *Dermacentor panamensis*, which was described from specimens collected in Central America, has long been confused with *Dermacentor halli*. In Mexico, what we consider bona fide specimens of *Dermacentor halli* have been recorded in the southern part of the country, in the states of Chiapas, Yucatán and Veracruz, but there remains the possibility that *Dermacentor panamensis* may also be found in this region. Until fresh specimens of both species become available for molecular and morphological analysis, we accept the Mexican distribution of *Dermacentor halli* as described herein.

Based on literature records, 11 species of *Dermacentor* are known from Mexico, which represents 31.4% of the total number of species (35) generally recognized worldwide. However, there are two species – *Dermacentor andersoni* and *Dermacentor latus* – whose occurrence in the country needs to be confirmed. *Dermacentor andersoni* is a species of the northern Nearctic, missing from most of the North American Southwest, so the record from Chiapas seems doubtful. Moreover, the record from Tamaulipas is based on nymphs, which can be difficult to accurately determine to species, and the record from Chihuahua is suspect because Chavarría (1941) states that his tick specimens may have been collected on sheep transiting customs in Ciudad Juárez. On the other hand, *Dermacentor latus* is known only from *Tapirus bairdii* in Chiapas (Cruz-Aldán et al. 2006). This is a little-studied tick that is also thought to be endangered (Mihalca et al. 2011), and for that reason its occurrence in Mexico requires confirmation. Among the other nine *Dermacentor* species, the most widespread is *Dermacentor albipictus* (26 Mexican states), followed by *Dermacentor nitens* (20), and *Dermacentor variabilis* (18). In contrast, *Dermacentor hunteri* and *Dermacentor imitans* are both known from only two Mexican states. *Dermacentor albipictus*, *Dermacentor dissimilis*, *Dermacentor halli*, *Dermacentor nitens* and *Dermacentor variabilis* all occur in both the Neotropical and Nearctic Zoogeographic Regions. *Dermacentor hunteri* and *Dermacentor occidentalis* are chiefly regarded as Nearctic species, while *Dermacentor imitans* is considered a Neotropical species. Guglielmone et al. (2014) classify *Dermacentor parumapertus* as a Nearctic tick, but we have found records from the southern Mexican state of Chiapas; these may represent a misidentification, so we cannot conclude that this species’ range extends into the Neotropics.

We have located records of *Dermacentor* species from all federal entities in Mexico except Morelos (Figure 3). The Mexican states with the largest number of localities in which *Dermacentor* ticks have been collected are Chiapas (17 localities), followed by Nuevo León (9 localities), and Hidalgo, Tamaulipas and Veracruz (8 localities each). In some cases there is a record for a state, but the collection locality is unknown (Guanajuato, Michoacán, Querétaro, Tlaxcala and Zacatecas).

Artiodactyl and perissodactyl mammals are common hosts of *Dermacentor* species. *Dermacentor albipictus* is usually associated with these large mammal hosts, although we found that this species can also be associated with rodents (*Liomys irroratus*, *Peromyscus boylii* and *Peromyscus maniculatus*). Unfortunately, however, no information is available concerning the stages of *Dermacentor albipictus* found on rodent hosts. *Dermacentor variabilis* is more of a generalist species, found on hosts as diverse as Carnivora and Lagomorpha. Somewhat surprisingly, in Mexico only *Dermacentor halli* and *Dermacentor hunteri* have been reported to parasitize humans. Mexican records of *Dermacentor imitans* are scarce and confined to Chiapas and Oaxaca, where this tick is associated with Artiodactyla.

Except for *Dermacentor andersoni*, *Dermacentor occidentalis* and *Dermacentor latus*, all Mexican *Dermacentor* species are represented in the CNAC. Even so, our understanding of the distribution and host relationships of this genus in Mexico is far from complete, and for that reason additional collections are urgently needed, so that we may better comprehend the biology, systematics, ecology, and zoogeography of this biomedically important genus.
